# MiRNA-Mediated Regulation in Osteoarthritis Across Joint Tissues, Including Translational Perspectives in Dogs

**DOI:** 10.3390/ani16060904

**Published:** 2026-03-13

**Authors:** Gabriella Guelfi, Camilla Capaccia, Vicente Francisco Ratto, Francesco Ciancabilla, David Forti, Federica Valeri, Domenico Caivano, Antonello Bufalari, Margherita Maranesi

**Affiliations:** Department of Veterinary Medicine, University of Perugia, 06126 Perugia, Italy; camilla.capaccia@dottorandi.unipg.it (C.C.); vicentefrancisco.rattovalderrama@dottorandi.unipg.it (V.F.R.); francesco.ciancabilla@studenti.unipg.it (F.C.); david.forti@dottorandi.unipg.it (D.F.); federica.valeri@dottorandi.unipg.it (F.V.); domenico.caivano@unipg.it (D.C.); antonello.bufalari@unipg.it (A.B.); margherita.maranesi@unipg.it (M.M.)

**Keywords:** osteoarthritis, miRNA, circulating miRNA, joint tissue crosstalk, canine osteoarthritis

## Abstract

Osteoarthritis (OA) is increasingly conceptualized as a whole-joint disorder, in which pathological processes across articular cartilage, subchondral bone, synovium, and periarticular tissues are tightly interconnected. Within this context, miRNAs have emerged as central post-transcriptional regulators capable of integrating mechanical, inflammatory, and metabolic cues at the network level. In human OA, extensive evidence links miRNA dysregulation to cartilage catabolism, impaired stress adaptation, inter-tissue communication, and the emergence of extracellular and circulating miRNA (cmiRNA) signatures with diagnostic and translational relevance. In contrast, miRNA research in canine OA remains limited, despite dogs developing a naturally occurring form of the disease that closely mirrors human OA in clinical presentation, joint pathology, and biomechanical drivers. To date, only a single pilot study has systematically investigated miRNA expression in spontaneous canine OA, underscoring both the feasibility of miRNA profiling and the substantial gaps that persist in tissue validation and functional characterization. This review critically synthesizes miRNA-mediated regulatory mechanisms in human OA and leverages this evidence to define research priorities in canine OA, where experimental validation remains limited. By focusing on shared molecular contexts rather than assumed equivalence, the review defines a comparative framework highlighting cmiRNAs as promising non-invasive translational targets.

## Simple Summary

Osteoarthritis (OA) is a common joint disease in both humans and dogs that causes pain, reduced mobility, and gradual loss of joint function. Although it has traditionally been viewed as a cartilage problem, OA actually involves the entire joint, including bone, synovium, and surrounding tissues that communicate with each other during disease progression. As life expectancy has increased in both humans and dogs, the prevalence of OA has risen substantially, making it an increasingly relevant health problem in aging populations. At the molecular level, epigenetic mechanisms help shape how joint tissues respond to mechanical stress, inflammation, and aging. Among these mechanisms, microRNAs (miRNAs) are small molecules that regulate how genes work inside cells and can also be released into body fluids such as blood and joint fluid. Growing evidence from human studies shows that miRNAs play a key role in joint inflammation, cartilage breakdown, bone remodeling, and cell survival. Because they can circulate in stable forms, miRNAs are also being investigated as potential non-invasive biomarkers of joint disease. Dogs naturally develop OA in a way that closely resembles the human condition, making them a valuable model for comparative research. However, miRNA studies in canine OA are still very limited. This review summarizes current knowledge on miRNAs in human OA and highlights how these findings can guide future research in dogs to improve diagnosis, monitoring, and ultimately treatment of osteoarthritis in both species.

## 1. Introduction

Osteoarthritis (OA) is a progressive, multifactorial joint disease driven by genetic susceptibility, mechanical loading, aging, and environmental factors [1]. OA is now viewed as a whole-joint disorder characterized by coupled structural and inflammatory remodeling across joint tissues [2,3].

Accumulating evidence indicates that epigenetic mechanisms play a central role in shaping gene-expression programs associated with OA progression. DNA methylation, histone modifications, and non-coding RNAs contribute to long-lasting alterations in cellular phenotype and tissue homeostasis within the joint. Among these regulatory layers, miRNAs have emerged as particularly relevant modulators of OA-related pathways, given their ability to fine-tune gene expression in a context-dependent manner and to coordinate complex biological networks rather than single molecular targets.

In recent years, extensive research has characterized miRNA dysregulation in human OA across joint compartments (articular cartilage, subchondral bone, synovium, and synovial fluid). These studies indicate that miRNAs contribute to extracellular matrix (ECM) remodeling, inflammatory signaling, mechanotransduction, and cell fate decisions, thereby contributing to tissue-specific pathology and joint-compartment crosstalk within the OA joint. Extracellular and cmiRNAs are discussed separately below as candidates for minimally invasive disease monitoring.

In contrast to the rapidly expanding human literature, miRNA research in canine OA remains limited, despite canine OA closely mirroring human OA in clinical presentation, joint pathology, and biomechanical drivers. This gap motivates a dedicated section on canine OA to define priority directions for translation.

This review synthesizes evidence on miRNA regulation in OA, emphasizing joint-compartment specificity, mechanobiology, inflammatory context, and extracellular/cmiRNAs as biomarkers and mediators of progression. Overall, it identifies mechanistic gaps and defines research priorities, with an explicit focus on canine OA.

To structure the human-focused sections, key studies identified through citation-network analysis were used to define three major domains: (a) cartilage catabolism, apoptosis, and autophagy; (b) subchondral bone, synovium, and periarticular crosstalk; and (c) extracellular circulating miRNAs as biomarkers. Despite extensive research on miRNA regulation in human OA, integration of compartment-specific regulation with extracellular miRNA signaling and translational relevance to spontaneous canine OA remains limited. This review addresses this gap by organizing evidence within a defined biological framework and examining whether regulatory patterns identified in human OA are conserved in spontaneous canine disease.

**Figure 1:**
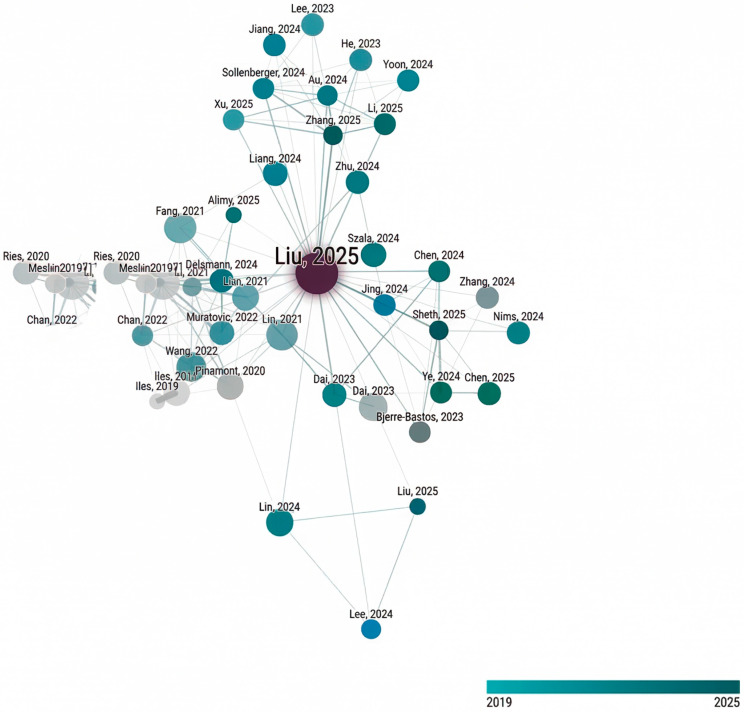
Citation network of key publications on miRNA involvement in osteoarthritis generated using the Connected Papers tool (https://www.connectedpapers.com). The network was constructed using the seed publication “Osteocyte-derived extracellular vesicles (EVs) mediate the bone-to-cartilage crosstalk and promote OA progression” [4]. Nodes represent individual publications, with node size proportional to citation influence and color indicating publication year. Edges denote citation relationships among studies. The network highlights a core group of highly interconnected publications that define major research trajectories in the field, including miRNA regulation of cartilage catabolism and cell fate, miRNA-mediated inter-tissue communication involving subchondral bone and synovium, and the emerging role of circulating and extracellular miRNAs as biomarkers.

Figure 1 shows the citation network generated using the Connected Papers tool (https://www.connectedpapers.com), with the seed article “Osteocyte-derived extracellular vesicles (EVs) mediate the bone-to-cartilage crosstalk and promote OA progression” [4]. The network informed the structural organization of Section 2, Section 3 and Section 4 and highlights the scarcity of comparable studies in canine OA, supporting the inclusion of a dedicated comparative section.

## 2. Literature Search Strategy and Selection Criteria

PubMed, Scopus, and Web of Science were searched to identify peer-reviewed studies on miRNA regulation in OA. The search covered January 2005–January 2026 and used combinations of the following terms: “osteoarthritis”, “microRNA” or “miRNA”, “circulating microRNA”, “extracellular vesicles”, “cartilage”, “subchondral bone”, “synovium”, and “canine osteoarthritis”, applying Boolean operators to refine retrieval.

Eligible records included original studies reporting miRNA expression and/or functional evidence in OA-relevant tissues or biofluids, with emphasis on extracellular and circulating miRNAs. Systematic reviews and meta-analyses were included when they supported the interpretation of primary findings. Records were excluded if they were not directly relevant to OA or did not provide functional insight.

To complement the primary database search and ensure inclusion of influential studies, citation-network analysis was performed using the Connected Papers tool (https://www.connectedpapers.com). The initial identification of relevant publications was therefore based on database retrieval (PubMed, Scopus, and Web of Science), while the citation-network analysis served as a complementary strategy to explore citation relationships among studies and to identify additional highly connected publications. The network was generated using the seed article “Osteocyte-derived extracellular vesicles (EVs) mediate the bone-to-cartilage crosstalk and promote OA progression” [4] and was used to identify highly connected and influential publications that informed the organization of the review and provided the basis for Figure 1.

The limited availability of canine-specific evidence was confirmed quantitatively. A PubMed search conducted in January 2026 using the terms “osteoarthritis AND (canine OR dog)” returned several hundred publications over the past five years, whereas the addition of “miRNA OR microRNA” yielded only one eligible original study, namely the pilot investigation by Yamazaki et al. [5] on circulating miRNAs in spontaneous canine OA. Accordingly, evidence from human OA was used as a reference framework, and the available canine data were examined to identify translational gaps and priorities for future investigation.

It should be noted that the human OA literature on miRNA regulation is substantially broader than the subset represented in Figure 1. The citation network was not intended to provide exhaustive coverage, but to identify highly connected and conceptually informative studies that define the main regulatory frameworks discussed in this review. Publications were therefore selected based on network connectivity, functional relevance, and their contribution to the current understanding of miRNA-mediated regulation in OA.

## 3. Molecular Mechanisms of OA Pathogenesis

OA is the most common musculoskeletal and joint disorder in both humans and dogs and is associated with chronic pain, reduced mobility, and progressive functional disability. The histopathology and pathogenetic mechanisms of canine OA closely overlap with those observed in humans, as do the main joint sites affected, knee, hip, shoulder, and elbow, and the nature of the pain, which appears to share similar neurophysiological underpinnings [6]. The onset and progression of OA in both species result from the interaction between genetic predisposition, biomechanical factors, and environmental influences [7]. Advanced age, obesity, body size, gender, and joint conformation are common OA risk factors [8], with mechanical factors, such as prior injuries, surgeries, excessive activity, or inactivity, also contributing [9]. Nutritional studies in dogs have shown that being overweight increases the risk of OA [10], highlighting how, similar to humans [11], obesity affects both biomechanical joint overload and systemic metabolic and inflammatory mechanisms. Furthermore, humans and companion dogs often share similar environments, lifestyles, physical activity levels, and even therapeutic strategies, including chronic administration of anti-inflammatory drugs, making spontaneous OA in dogs an important translational model for human medicine.

**Figure 2:**
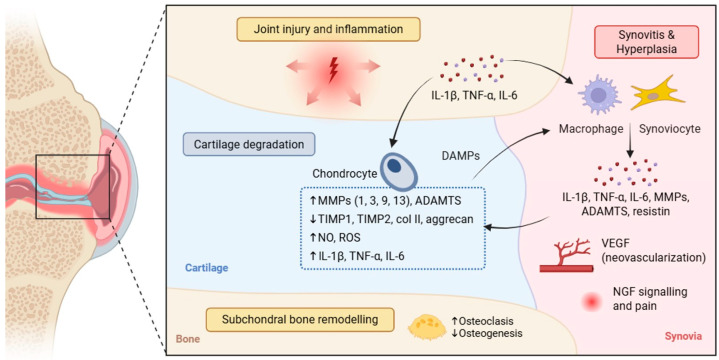
Pathophysiological mechanisms involved in OA progression. Mechanical stress, injury, and inflammatory stimuli promote the release of proinflammatory cytokines (IL-1β, TNF-α, IL-6) within the joint. This leads to chondrocyte activation and increased production of matrix-degrading enzymes (MMPs and ADAMTS), nitric oxide (NO), and reactive oxygen species (ROS), while reducing the production of tissue inhibitors of metalloproteinases (TIMP)-1 and TIMP-2. These processes result in the degradation of extracellular matrix (ECM) components, including type II collagen and aggrecan. Degradation products act as damage-associated molecular patterns (DAMPs), further activating synovial macrophages and synoviocytes, which release additional cytokines, catabolic mediators, and proangiogenic factors such as VEGF, promoting synovitis and neovascularization. Furthermore, nerve growth factor (NGF) signaling in inflamed synovial tissue contributes to nociceptor sensitization and osteoarthritis-associated pain. In subchondral bone, the balance between osteogenesis and osteoclast activity is altered. This is accompanied by bone hyperplasia, hardening, and osteophyte formation.

OA is characterized by degeneration and progressive remodeling of the entire synovial joint, characterized by metabolic alterations in the articular cartilage, subchondral bone remodeling, and synovial inflammation [12]. Articular cartilage consists of ECM that is primarily made up of water, collagen, proteoglycans (PGs), and chondrocytes, which are the only cells present. Under physiological conditions, these cells maintain tissue homeostasis by balancing the synthesis and degradation of ECM components [13]. However, in OA, this balance is disrupted by mechanical stress, trauma, and aging-related inflammatory processes (inflammaging), which increase the production of proinflammatory cytokines, particularly interleukin (IL)-1β, tumor necrosis factor (TNF)-α, and IL-6 [14]. These molecules stimulate chondrocytes to produce, in an autocrine and paracrine manner, additional cytokines and matrix degradative enzymes, such as metalloproteinases (MMP-1, MMP-3, MMP-9, MMP-13) and aggrecanases (ADAMTS-4 and ADAMTS-5), while simultaneously reducing the synthesis of key ECM components, including type II collagen and PGs [15], finally promoting cartilage degradation. Chondrocyte activation is also associated with increased expression of inducible nitric oxide synthase (iNOS), which leads to the production of nitric oxide (NO) and reactive oxygen species (ROS). These substances cause oxidative stress, cell damage, and chondrocyte apoptosis, thereby sustaining a self-perpetuating inflammatory cycle [16]. In the early stages of disease, chondrocytes attempt to compensate for the loss of ECM by increasing the synthesis of its components and proliferating. However, as the disease progresses, cell loss, chondrocyte senescence, and structural alterations to the ECM prevail, leading to increased water content, PG depletion, and weakening of the collagen network. This progressive destruction of cartilage leads to remodeling of the subchondral bone, which is typically characterized by sclerosis and osteophyte formation [13]. Synovial inflammation (synovitis) represents another key contributor to OA progression. The synovial membrane develops chronic synovitis, characterized by hyperplasia of the synovial layer, infiltration of inflammatory cells, and neovascularization mediated by pro-angiogenic factors, such as VEGF [17]. Activated synoviocytes and synovial macrophages produce high levels of pro-inflammatory cytokines (IL-1β, IL-6, and TNF-α), matrix-degrading enzymes (MMPs and ADAMTS), adipokines such as resistin, and other catabolic molecules, which diffuse into the synovial fluid and articular cartilage, further promoting ECM degradation [18]. In turn, ECM degradation products act as damage-associated molecular patterns (DAMPs), further sustaining synovial macrophage activation and establishing a self-perpetuating cycle of inflammation and tissue destruction [19]. Synovial inflammation is also closely associated with peripheral sensitization mediated by nerve growth factor (NGF) signaling [20,21]. In inflamed joint tissues, particularly in the synovium, the expression of NGF is upregulated by pro-inflammatory cytokines such as IL-1β and TNF-α, contributing to nociceptor sensitization and the development of OA-associated pain (Figure 2).

## 4. Biological Basis of miRNA Regulation in OA

Cellular homeostasis within joint tissues relies on finely coordinated regulatory mechanisms that extend beyond transcriptional control. In this context, miRNAs constitute a class of regulatory non-coding RNAs that fine-tune gene expression through post-transcriptional mechanisms, primarily by interacting with partially complementary sequences in target messenger RNAs, thereby modulating translation efficiency or transcript stability. Rather than functioning as binary regulators, miRNAs exert subtle yet cumulative effects on multiple targets simultaneously, enabling robust control of complex gene networks in a context-dependent manner [22].

MiRNA biogenesis is a tightly regulated, multistep process that confers an additional layer of control over gene expression. Primary miRNA transcripts are processed in the nucleus by the Drosha–DGCR8 microprocessor complex to generate precursor miRNAs, which are subsequently exported to the cytoplasm and cleaved by Dicer to produce mature miRNA duplexes. Incorporation of the guide strand into the RNA-induced silencing complex (RISC) enables sequence-specific repression of target transcripts, allowing miRNAs to dynamically adjust cellular responses to developmental, mechanical, and inflammatory cues [22].

Joint tissues, including articular cartilage, synovium, subchondral bone, and periarticular connective structures, are characterized by low cellular turnover and prolonged exposure to biomechanical and biochemical stressors. In these tissues, miRNA-mediated regulation plays a critical role in maintaining the balance between anabolic and catabolic processes by modulating pathways involved in ECM synthesis, cell survival, differentiation, and inflammatory signaling [23]. This regulatory flexibility is especially relevant in tissues with limited regenerative capacity, where sustained molecular adaptation is required to preserve structural integrity.

This post-transcriptional layer of regulation is therefore well suited to sustain long-term tissue integrity while permitting adaptive responses to environmental changes [24].

Mechanical loading represents a defining feature of joint biology and a major determinant of miRNA expression profiles, particularly in tissues exposed to repetitive or excessive biomechanical stress [25].

This mechanosensitive regulation links joint biomechanics to post-transcriptional control across multiple joint cell types [26]. Through this mechanism, miRNAs contribute to load-dependent tissue adaptation, whereas persistent or aberrant mechanical stress may drive maladaptive miRNA responses that favor degeneration and structural remodeling [25].

Inflammatory mediators further shape miRNA expression in joint tissues. Pro-inflammatory cytokines such as IL-1β and TNF-α regulate specific miRNAs that, in turn, modulate key signaling pathways including NF-κB, MAPK, and TGF-β. By integrating inflammatory signals with structural and metabolic pathways, miRNAs function as molecular nodes that coordinate immune activation with tissue remodeling processes [27]. Importantly, miRNA function in joint tissues is highly context-dependent. The regulatory outcome of a given miRNA varies across cell types, differentiation states, mechanical environments, and inflammatory milieus. Such properties make miRNAs particularly suited to modulate disease-relevant regulatory programs in OA [28].

## 5. MiRNAs in Human Articular Cartilage: Catabolism, Apoptosis, and Autophagy

In human articular cartilage, OA is characterized by a progressive imbalance between tissue maintenance and degeneration, driven by coordinated alterations in catabolic activity, cell survival, and stress-adaptive mechanisms. In this context, catabolism refers to the excessive degradation of the ECM mediated by proteolytic enzymes and inflammatory signaling, leading to the loss of cartilage structural integrity. Concomitantly, apoptosis contributes to disease progression through the gradual loss of viable chondrocytes, reducing the cellular capacity required for matrix maintenance and repair. Under physiological conditions, autophagy acts as a protective mechanism that allows chondrocytes to adapt to mechanical, metabolic, and oxidative stress by preserving cellular homeostasis. However, in OA cartilage, autophagic activity is frequently impaired, limiting stress adaptation and favoring a shift toward apoptotic pathways. Together, the dysregulation of catabolism, apoptosis, and autophagy defines a pathogenic triad that undermines cartilage resilience and accelerates degeneration in human OA. MiRNAs emerge as key post-transcriptional regulators of these interconnected processes, integrating inflammatory, mechanical, and metabolic cues that shape chondrocyte fate and cartilage homeostasis.

In OA, miRNAs operate as post-transcriptional regulators that can couple biomechanical and biochemical inputs to coordinated shifts in extracellular matrix turnover, inflammatory signaling, and cell fate decisions.

A major driver of cartilage dysfunction in OA is the conversion of mechanical cues into molecular responses. Chondrocytes possess a broad mechanosensitive program (“mechanome”) in which activation of mechanosensitive ion channels such as TRPV4 [29] and PIEZO1 [30] induces distinct transcriptional outputs, supporting the concept that the nature, amplitude, and duration of mechanical stimulation can generate qualitatively different downstream states in cartilage cells [29]. Because multiple mechanoresponsive pathways converge on inflammatory mediators and matrix-degrading enzymes, dysregulated mechanotransduction provides a plausible upstream context in which miRNA-mediated post-transcriptional control can reinforce catabolic polarization in cartilage.

Catabolism is further amplified by inter-tissue signaling mechanisms that directly target chondrocyte homeostasis. Mechanistic evidence indicates that osteoclast-derived exosomes can transfer miRNA cargoes to chondrocytes, reducing cartilage resistance to matrix degeneration and promoting pro-degradative programs through repression of protective inhibitors of matrix breakdown (e.g., TIMP-2/TIMP-3), thereby functionally linking bone remodeling to cartilage catabolism via miRNA-mediated communication [31]. Consistently, osteoclast-derived exosomal miRNAs have been shown to accelerate cartilage catabolism and suppress anabolic activity by modulating the TGF-β axis through TGF-β1/Smad2-related signaling, highlighting a mechanistically defined pathway through which miRNA transfer can shift the anabolic–catabolic balance in chondrocytes [32]. In parallel, exosomal miRNA-dependent regulation of Smad2 has been implicated in promoting chondrocyte hypertrophic differentiation, a phenotype associated with impaired cartilage maintenance and enhanced matrix remodeling [33]. Among these regulatory miRNAs, miR-30a/b has been observed to be upregulated in OA cartilage, where it inhibits chondrocyte proliferation and differentiation while promoting inflammation and ECM [34]. In particular, miR-30a/b reduces the expression of type II collagen (COL2A1) and aggrecan and increases IL-1β expression [35]. Other studies have found that miR-30b-5p increases MMP-13, active caspase-3, and TNF-α levels in chondrocytes, thereby reinforcing ECM breakdown [36]. Together, these studies support the view that chondrocyte catabolism in OA is shaped not only by intrinsic regulatory changes but also by extracellular miRNA signals originating from other joint compartments.

Apoptosis represents a complementary axis of cartilage deterioration, as loss of viable chondrocytes compromises the tissue’s already limited capacity for long-term matrix renewal. In this regard, oxidative stress is increasingly recognized as a key determinant of chondrocyte survival and regenerative competence. Experimental evidence in adult mice demonstrates that mitigation of reactive oxygen species can enhance cartilage regeneration by reducing apoptosis of cartilage progenitor cells, indicating that redox status is a modifiable upstream variable controlling survival programs in cartilage-relevant cell populations [37]. Although this work is not OA-specific and is performed in an experimental regeneration setting, it provides a mechanistic rationale for interpreting oxidative stress–responsive miRNA networks as potential modulators of apoptosis susceptibility within cartilage under chronic joint stress.

At the regulatory level, miRNA effects in cartilage-relevant pathways can also be shaped by epigenetic mechanisms that control miRNA expression and availability. The histone methyltransferase SETDB1 was shown to modulate osteogenic responses under mechanical unloading by suppressing miR-212-3p expression via promoter-associated H3K9 methylation, establishing a defined epigenetic–miRNA axis that is responsive to mechanical context [38]. While this study is centered on osteogenic differentiation, it strengthens the broader concept developed in this review: mechanically sensitive epigenetic regulation can influence miRNA levels and thereby alter downstream tissue remodeling programs across the osteochondral unit.

Finally, autophagy constitutes a central adaptive mechanism that supports chondrocyte survival under nutrient limitation, oxidative stress, and mechanical overload. In OA, defective or insufficient autophagic responses are frequently discussed as permissive conditions that favor apoptosis and catabolic polarization. In the context of the studies summarized above, the convergence of mechanotransduction, oxidative stress, and extracellular miRNA transfer provides a coherent framework in which miRNA-mediated regulation may play a significant role in the balance between autophagy and apoptosis, as well as the stabilization of long-term degenerative processes in cartilage.

**Figure 3:**
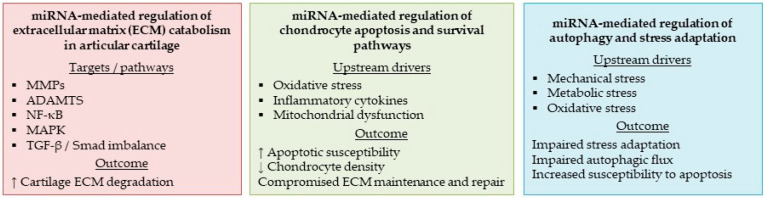
MiRNA-mediated regulation of cartilage homeostasis in human OA. This figure summarizes the integrated roles of miRNAs in key processes driving cartilage degeneration in human OA. The red block highlights miRNA control of ECM catabolism through regulation of matrix-degrading enzymes (MMPs, ADAMTS) and major signaling pathways (NF-κB, MAPK, and TGF-β/Smad imbalance), promoting ECM degradation. The green block depicts miRNA modulation of chondrocyte survival and apoptosis in response to oxidative stress, inflammatory cytokines, and mitochondrial dysfunction, increasing apoptotic susceptibility and compromising ECM maintenance. The blue block illustrates miRNA regulation of autophagy and stress adaptation (including AMPK–mTOR–related pathways), where impaired stress responses reduce cellular resilience and favor a shift toward apoptosis.

These findings support a regulatory model in which cartilage degeneration in OA is governed by coordinated networks linking mechanotransduction, redox-dependent survival pathways, and extracellular miRNA signaling from adjacent joint compartments. However, the relative contribution of individual miRNAs and their consistency across disease stages remain incompletely defined. These regulatory interactions indicate that cartilage-associated miRNAs reflect tissue state and contribute to the control of catabolic activity, apoptosis, and stress response pathways during disease progression. Figure 3 summarizes these relationships, illustrating miRNA-associated coordination of chondrocyte survival and catabolic processes in human articular cartilage. Comparable regulatory patterns in canine articular cartilage remain unexplored, as tissue-level miRNA studies in spontaneous canine OA are currently unavailable.

## 6. miRNAs in Human Subchondral Bone, Synovium, Periarticular Tissues, and Synovial Fluid

In the OA joint, pathological remodeling of subchondral bone, synovial inflammation, cartilage degeneration, and periarticular tissue alterations are tightly interconnected. This section extends the cartilage-focused view to subchondral bone, synovium, periarticular tissues, and synovial fluid [39]. Human-based studies increasingly indicate that miRNA dysregulation extends beyond articular cartilage, involving subchondral bone, synovium, periarticular tissues, and synovial fluid, where miRNAs act both as intracellular regulators and as extracellular mediators of inter-tissue communication.

In human subchondral bone, altered miRNA expression has been consistently associated with abnormal bone remodeling, osteoclast activation, and impaired osteoblast–osteocyte coupling. Histomorphometric and molecular analyses of OA femoral heads and knee specimens demonstrate that regions of sclerosis and cartilage loss are characterized by distinct miRNA signatures regulating osteogenic differentiation, ECM turnover, and osteoclastogenesis [40,41]. In particular, miRNAs targeting components of the TGF-β/Smad pathway, RANKL signaling, and mechanosensitive regulators have been implicated in driving uncoupled subchondral bone remodeling and altered mechanoadaptation [42]. Among these, members of the miR-30 family (miR-30a, miR-30b, miR-30c, and miR-30d) have been shown to inhibit osteogenesis by targeting key osteogenic regulators such as Smad1 and RUNX2 [43], further supporting the role of miRNA-mediated mechanisms in subchondral bone remodeling. These findings support a spatial and functional link between miRNA-mediated bone dysregulation and overlying cartilage degeneration in human OA.

The synovium represents a second major compartment in which miRNAs contribute to disease progression. Analyses of synovial tissue obtained from patients with knee and hip OA reveal dysregulation of miRNAs involved in inflammatory signaling, fibroblast-like synoviocyte proliferation, and macrophage activation [39]. These synovial miRNAs modulate cytokine networks, including IL-6, TNF, and NF-κB pathways, as well as the expression of matrix-degrading enzymes, thereby indirectly promoting cartilage catabolism and sustaining chronic low-grade inflammation within the joint.

Synovial fluid constitutes an integrated molecular reservoir reflecting contributions from cartilage, subchondral bone, synovium, and periarticular tissues. Multiple human studies have identified distinct miRNA profiles in synovial fluid that correlate with radiographic severity, biochemical markers of cartilage degradation, and mechanical stress exposure [44]. Importantly, miRNAs detected in synovial fluid are predominantly present in a stabilized extracellular form, frequently associated with EVs or protein complexes, supporting their suitability as minimally invasive biomarkers for joint-level pathological processes. Consistent with this compartmental integration, periarticular structures (e.g., ligaments, capsule, infrapatellar fat pad) may also contribute to the extracellular miRNA pool through stress- and inflammation-responsive signaling.

**Figure 4:**
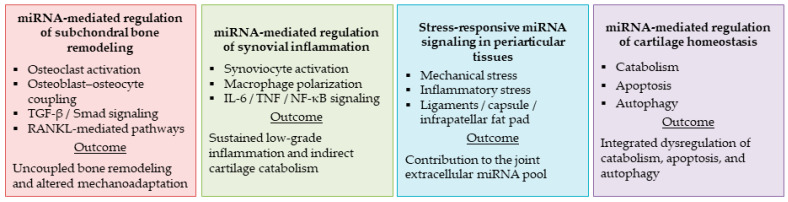
MiRNA-mediated inter-tissue regulation across the OA joint. This figure summarizes the compartment-specific and integrated roles of miRNAs in coordinating molecular and cellular processes across the osteoarthritic joint. The red block highlights miRNA-mediated regulation of subchondral bone remodeling, including effects on osteoclast activity, osteoblast–osteocyte coupling, and mechanosensitive signaling pathways. The green block depicts synovial miRNA control of inflammatory signaling, synoviocyte activation, and macrophage polarization, which sustains low-grade inflammation and indirectly promotes cartilage catabolism. The blue block illustrates stress-responsive miRNA signaling in periarticular tissues (ligaments, joint capsule, and infrapatellar fat pad) and their contribution to the extracellular miRNA pool within the joint. The violet block integrates miRNA-mediated regulation of cartilage homeostasis, encompassing catabolic activity, apoptotic susceptibility, and autophagy as interconnected processes driving cartilage degeneration in OA.

These observations extend the role of miRNAs from intracellular regulators to mediators of coordinated interactions between joint compartments. Experimental evidence shows that subchondral bone cells, including osteocytes and osteoclasts, release EVs enriched in specific miRNA cargoes that can be transferred to chondrocytes, where they influence anabolic–catabolic balance, hypertrophic differentiation, and cell survival [32,33]. This evidence supports EV-mediated regulatory communication; however, direct in vivo validation and the extent of these interactions across disease stages remain limited. Comparable investigations in canine OA have not yet been performed, limiting current understanding of miRNA regulation across joint compartments in spontaneous disease. Figure 4 summarizes EV-associated miRNA transfer and inter-compartmental signaling within the OA joint.

Collectively, human evidence positions miRNAs as central integrators of mechanical loading, subchondral bone remodeling, synovial inflammation, and cartilage degeneration. Their compartment-specific dysregulation, combined with their extracellular trafficking through synovial fluid and vesicles, underscores their dual role as drivers of disease progression and as promising molecular indicators of joint-wide pathology.

Accordingly, the main human studies identifying compartment-specific miRNA signatures in subchondral bone, synovium, periarticular tissues, and synovial fluid, together with their principal molecular targets and biological effects, are summarized in Table 1, which provides a structured integration of compartment-specific miRNA dysregulation and inter-compartmental regulatory interactions.

## 7. Circulating miRNAs in Human OA Pathology

CmiRNAs have emerged as promising molecular indicators of OA, as they reflect the integrated molecular activity across joint tissues rather than isolated alterations within individual compartments. Unlike tissue-restricted miRNA profiles, cmiRNAs detected in biological fluids represent composite regulatory signals derived from subchondral bone, cartilage, synovium, and periarticular tissues, supporting their relevance as systemic indicators of joint pathology [44].

Human studies indicate that cmiRNA profiles are sensitive to whole-joint pathological changes and mechanical stress exposure. In particular, EV-associated miRNAs represent a stable circulating fraction capable of preserving biologically meaningful information reflecting tissue-level regulation [52]. Importantly, cmiRNAs are not exclusively confined to EVs, but can also be detected in association with protein complexes, such as Argonaute proteins [53] and lipoproteins [54], which contribute to their stability and persistence in biological fluids [55]. Mechanistic evidence demonstrates that bone-resident cells, including osteocytes and osteoclasts, actively contribute to the extracellular miRNA pool through EV-mediated release, thereby linking subchondral bone remodeling to downstream effects on cartilage homeostasis [4,32,33].

Several studies show that osteoclast- and osteocyte-derived EV-associated miRNAs regulate key chondrocyte processes, including anabolic–catabolic balance, hypertrophic differentiation, and cell survival. Although these investigations primarily address intercellular communication within the joint, they also support the concept that disease-associated miRNA cargoes generated in joint tissues can reach extracellular compartments and ultimately be detected in biological fluids [4,33]. In parallel, experimental evidence indicates that mechanical loading, matrix stiffness, and mechanotransduction pathways modulate EV release and miRNA cargo composition in bone- and cartilage-related cells, providing a mechanistic basis for the dynamic modulation of cmiRNA profiles in response to joint biomechanics [33].

On this mechanistic basis, increasing attention has been directed toward the use of cmiRNAs as biomarkers of whole-joint pathology in OA. From a diagnostic perspective, cmiRNAs detected in synovial fluid and blood have been reported to correlate with radiographic severity, biochemical markers of cartilage degradation, and joint mechanical burden in human OA, suggesting that they function as dynamic indicators of disease activity rather than static markers of structural damage [44]. Importantly, the biomarker value of cmiRNAs appears to rely more on multi-miRNA signatures than on individual molecules, as such signatures better capture the complexity of joint-wide molecular dysregulation.

For a cmiRNA to be considered a reliable biomarker of a specific pathology, it should reflect the molecular state of the affected tissue rather than representing a nonspecific systemic signal. Accordingly, the identification of candidate cmiRNA biomarkers should ideally follow a tissue-first strategy, in which differentially expressed miRNAs are first identified within the damaged tissue and subsequently evaluated in circulation, typically in serum or plasma, to determine whether they are also dysregulated in affected individuals [56]. This tissue-to-circulation validation framework strengthens biological plausibility and supports the interpretability of cmiRNA signatures, particularly when EVs mediate the release and stabilization of tissue-derived miRNAs into biological fluids [57]. Beyond their diagnostic relevance, miRNAs that are consistently dysregulated at both tissue and circulating levels may also represent attractive therapeutic targets. Because miRNAs regulate gene expression at the post-transcriptional level and often control entire gene networks, their modulation offers the possibility to influence complex pathological pathways rather than single downstream effectors. When a miRNA is pathologically overexpressed, its activity can be inhibited using antisense oligonucleotides, commonly referred to as anti-miRs, which are chemically modified single-stranded nucleic acids designed to bind and neutralize mature miRNAs, thereby preventing their interaction with target transcripts [58].

Conversely, miRNAs that are downregulated in disease contexts can be restored through the delivery of synthetic miRNA mimics, which functionally replace the endogenous miRNA and re-establish physiological regulatory control over target gene expression [59]. Both anti-miR-based inhibition and miRNA replacement strategies therefore constitute forms of gene-regulatory therapy, and several miRNA-based approaches have already progressed into clinical development in non-musculoskeletal diseases, demonstrating the feasibility of this therapeutic paradigm [60].

From a comparative standpoint, cmiRNAs offer potential advantages over conventional protein biomarkers by reflecting upstream regulatory events rather than downstream tissue damage. Because miRNA expression and release are dynamically modulated by mechanical, inflammatory, and metabolic cues, cmiRNA profiles may provide early indications of disease activity or changes in joint biomechanics before irreversible structural alterations become apparent [4,33]. Reviews integrating mechanistic and translational evidence further highlight the potential of targeting epigenetic and miRNA-related pathways, including EV-mediated signaling, to interfere with pathological bone–cartilage crosstalk in OA [61].

Several challenges must be addressed before cmiRNAs can be implemented in clinical practice, including biological variability, differences in sample collection and processing, limited analytical standardization, and the difficulty of distinguishing joint-derived signals from systemic background. These limitations support the use of standardized protocols and multi-miRNA panels rather than single candidates [62]. Human studies indicate that circulating miRNAs reflect regulatory processes occurring across cartilage, subchondral bone, synovium, and periarticular tissues, supporting their potential application in disease stratification and longitudinal monitoring; however, their tissue specificity and causal relationships remain to be fully established. In canine OA, circulating miRNA evidence is limited to a single exploratory study, and validation across joint tissues and circulation remains unresolved.

## 8. MiRNAs in Canine OA and Emerging Translational Challenges

Evidence on miRNA regulation in spontaneous canine OA remains extremely limited, with only one original study reporting circulating miRNA expression. In contrast, miRNA dysregulation in human OA has been characterized across cartilage, subchondral bone, synovium, synovial fluid, and circulation. This disparity persists despite the fact that dogs develop a naturally occurring form of OA that closely parallels the human disease in clinical presentation, joint pathology, and biomechanical drivers. Accordingly, canine OA provides a relevant comparative model for investigating miRNA-mediated regulation in naturally occurring joint degeneration. The following section summarizes the available evidence and identifies priorities for standardization, tissue-to-circulation validation, and comparative study design.

To date, only one original peer-reviewed study has comprehensively investigated miRNA expression in dogs affected by spontaneous OA. Yamazaki et al. [5] performed a pilot investigation integrating small RNA sequencing and RT–qPCR validation to profile miRNA expression across multiple biological matrices, including synovial tissue, synovial fluid, and serum, in dogs with naturally occurring OA. This study represents the first and, at present, the only experimental analysis directly linking miRNA dysregulation to spontaneous OA in the canine species.

Importantly, the work by Yamazaki et al. [5] demonstrated that OA-associated miRNA alterations are detectable not only at the tissue level but also in extracellular compartments, supporting the concept that cmiRNAs may reflect joint-specific pathological processes in dogs. However, the exploratory nature of the study, the limited cohort size, the cross-sectional design, and the absence of functional validation collectively underscore the preliminary status of the available evidence. To date, no independent replication studies, longitudinal analyses, or mechanistic investigations addressing the functional role of specific miRNAs in canine OA progression have been reported.

Beyond this pilot investigation, references to miRNA-based diagnostics or therapeutic strategies in canine OA are largely confined to non–peer-reviewed sources, including conference communications, industry communications, or general discussions within broader reviews of miRNA biology in dogs. Comprehensive surveys of miRNA expression studies in the canine species confirm that, although miRNA research is expanding across multiple pathological contexts, robust experimental data specifically addressing OA remain scarce [63]. These analyses further highlight substantial heterogeneity in study design, biological matrices, and analytical pipelines, which currently limits cross-study and comparative interpretation.

This limited evidence contrasts with the extensive human literature, in which miRNA dysregulation has been documented across cartilage, subchondral bone, synovium, synovial fluid, and circulation, highlighting a major translational gap. This imbalance is particularly striking given the spontaneous nature of canine OA and its close resemblance to the human condition. From a comparative perspective, the dog offers a unique opportunity to investigate miRNA-mediated regulatory mechanisms within a naturally evolving joint pathology, avoiding some of the limitations inherent to induced experimental models.

**Figure 5:**
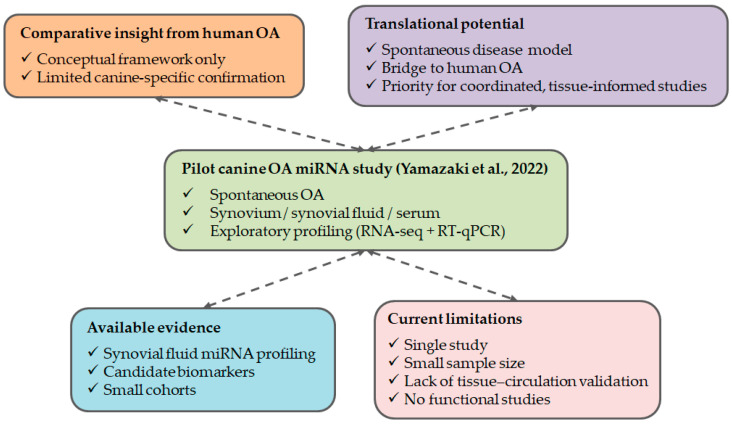
Current landscape of miRNA research in canine OA and translational relevance. This figure summarizes the current state of evidence on miRNAs in canine OA, highlighting the only original study to date that systematically investigated miRNA expression in spontaneous canine OA [5], using RNA-seq and RT-qPCR in synovium, synovial fluid, and serum. The upper panels illustrate the conceptual framework derived from human OA research and the comparative value of the dog as a spontaneous disease model bridging veterinary and human medicine. The lower panels depict the present evidence base, which remains largely exploratory and limited by small cohort sizes, reliance on a single study, lack of tissue-to-circulation validation, and absence of functional analyses. Dashed arrows denote conceptual and directional relationships, emphasizing that current translational inferences are primarily based on biological reasoning and comparison with human OA rather than consolidated experimental validation in the canine species.

Collectively, the current literature indicates that miRNA research in canine OA remains at an early, exploratory stage, dominated by a single pilot study and lacking systematic validation. This situation highlights the need for coordinated research efforts incorporating standardized tissue sampling, parallel analysis of joint compartments and circulating fluids, and functional evaluation of candidate miRNAs. Expanding miRNA research in canine OA holds significant promise not only for advancing veterinary diagnostics and disease monitoring but also for strengthening translational frameworks aimed at understanding miRNA-mediated regulation of joint degeneration across species (Figure 5).

## 9. Conclusions

OA is a whole-joint disease driven by coordinated structural, inflammatory, and mechanical alterations across multiple tissues. Within this context, miRNAs function as post-transcriptional regulators that integrate intracellular signaling and contribute to inter-compartmental communication within the joint.

In human OA, miRNA dysregulation has been documented across cartilage, subchondral bone, synovium, periarticular tissues, synovial fluid, and circulation. These regulators influence extracellular matrix turnover, cell survival, mechanotransduction, inflammatory pathways, and stress response mechanisms, consistent with coordinated regulation across joint compartments. The detection of circulating miRNAs further supports their role in inter-tissue signaling.

In contrast, evidence in canine OA remains limited despite strong clinical and pathological similarities with human disease. This disparity highlights the relevance of the dog as a comparative model for investigating miRNA regulation in naturally occurring OA. Expanding tissue-level and circulating miRNA studies in canine OA is required to support biomarker development and to clarify regulatory relationships across joint compartments. By integrating human and canine evidence within a unified biological framework, this review defines current knowledge boundaries and establishes a structured comparative framework. This framework represents a critical prerequisite for translating miRNA biology into clinically actionable biomarkers and therapeutic strategies in both veterinary and human OA. A comparative overview of miRNA evidence across joint compartments in human and canine OA is summarized in Table 2, highlighting the marked imbalance between extensive human evidence and the near absence of tissue-level validation in spontaneous canine OA.

## 10. Key Translational Gaps and Priority Research Directions

The growing literature on miRNA involvement in human OA has clarified multiple regulatory pathways; however, translational uncertainties persist.

Much of the available evidence derives from heterogeneous study designs, predominantly cross-sectional, which inherently limit temporal resolution and causal inference [64]. Second, substantial methodological differences across cohorts, analytical platforms, and miRNA quantification pipelines complicate direct comparability [64]. Third, standardization issues in sample collection, processing, and normalization strategies further reduce inter-study coherence and hinder evidence synthesis across the literature. Collectively, these limitations compromise reproducibility and impede cumulative knowledge building in the field. In parallel, tissue-to-circulation validation remains particularly underdeveloped. The extent to which circulating miRNA signatures faithfully mirror joint-specific dysregulation is unclear, as paired tissue and serum analyses within the same individuals are rare. Consequently, the biological origin of circulating signals remains uncertain, especially given well-documented pre-analytical and analytical variability in cmiRNA studies [65]. From a comparative standpoint, the imbalance between human and canine evidence is striking. Whereas human OA has been profiled across cartilage, subchondral bone, synovium, synovial fluid, and systemic circulation, spontaneous canine OA has been explored in only a single exploratory study, without independent replication, longitudinal assessment, or integrated tissue-level validation. This disparity constrains the development of robust comparative frameworks capable of distinguishing conserved regulatory networks from species-specific adaptations. Importantly, although a large proportion of canine miRNAs (~84%) show sequence homology to human counterparts, sequence conservation alone does not imply equivalence in tissue-specific expression patterns, target interactions, or disease-context regulatory functions [66]. Progress in the field will require coordinated study designs capable of directly linking molecular findings to joint pathology. Parallel profiling of cartilage, subchondral bone, synovium, and circulation within the same individuals is essential to determine whether circulating miRNA signatures genuinely reflect tissue-level dysregulation. Longitudinal and stage-stratified cohorts are needed to distinguish early drivers of disease from late secondary changes and to clarify whether specific miRNAs are associated with initiation, progression, or chronic remodeling. Finally, the adoption of standardized methodological workflows spanning pre-analytical handling, RNA isolation, quantification platforms, and normalization strategies is necessary to improve reproducibility and enable meaningful comparison across independent datasets. Without methodological harmonization, miRNA research risks remaining confined to descriptive profiling; rigorous standardization is required to establish causal mechanisms and enable meaningful translational integration in OA biology.

## 11. Expert Opinion

MiRNAs function as integrative regulators of OA biology, reflecting the combined mechanical, inflammatory, and metabolic state of the joint. Their clinical value is unlikely to reside in single molecules, but rather in coordinated multi-miRNA signatures capable of capturing whole-joint dysregulation. However, translational implementation requires rigorous cross-cohort validation and clear linkage between circulating profiles and tissue-level pathology. Beyond their biomarker potential, miRNAs represent regulatory nodes capable of modulating interconnected molecular pathways without direct genomic manipulation, offering a systems-level therapeutic perspective. From a comparative standpoint, spontaneous canine OA provides a valuable framework for biologically anchoring circulating miRNA findings to joint pathology in a naturally occurring disease context. Integrating tissue and circulating analyses in dogs may therefore represent a critical step toward the translational validation of miRNA-based strategies in osteoarthritis.

## Figures and Tables

**Table 1:** Compartment-specific miRNA dysregulation across the OA joint. The table summarizes key miRNAs identified in human OA across subchondral bone, synovium, periarticular tissues, and synovial fluid, together with their principal molecular targets and pathophysiological relevance. Tissue-specific and extracellular (circulating and EV-associated) miRNA signatures are included, highlighting their contribution to joint-wide regulatory networks and inter-tissue crosstalk. References are reported as reference numbers corresponding to the reference list. Reported associations reflect context-dependent regulatory interactions rather than single linear causal relationships.

Joint Compartment	Key miRNAs/Class	Main Targets/Pathways	OA Pathophysiological Relevance	Reference
Subchondral bone	miR-212-3p	TGF-β1/Smad signaling	Associated with osteoclast-related signaling and dysregulated subchondral bone remodeling.	[38]
Subchondral bone	let-7a-5p	Smad2	Associated with enhanced chondrocyte hypertrophic differentiation and cartilage catabolic processes.	[45]
Subchondral bone	Multiple miRNAs	RANKL–osteoclastogenesis axis	Implicated in dysregulated bone resorption and subchondral sclerosis.	[41,46]
Subchondral bone	Mechanosensitive miRNA signature	TGF-β/mechanotransduction pathways	Associated with altered bone adaptation to mechanical loading.	[42]
Synovium	Inflammation-associated miRNAs	IL-6, TNF, NF-κB signaling	Associated with sustained synovial inflammation and cytokine-driven joint catabolic activity.	[47,48]
Synovium	ECM-regulatory miRNAs	MMPs, ADAMTS	Indirectly associated with cartilage extracellular matrix degradation.	[48]
Synovial fluid	cmiRNAs	Multicompartment origin (bone, cartilage, synovium)	Correlation with radiographic severity and cartilage damage	[49]
Synovial fluid	EV-associated miRNAs	Post-transcriptional regulation in chondrocytes	Implicated in inter-tissue communication within the OA joint.	[4,50]
Periarticular tissues	Stress-responsive miRNAs	Oxidative stress and apoptotic pathways	Associated with tissue stress responses, resilience, and regenerative potential.	[51]
Whole joint (EV-mediated)	Bone-derived miRNA cargo	Anabolic–catabolic balance in chondrocytes	Implicated in bone-to-cartilage molecular crosstalk	[4,31,50]

**Table 2:** Comparative overview of miRNA evidence across joint compartments in human and canine OA.

Joint Compartment	Human OA Evidence	Canine OA Evidence
Articular cartilage	Extensive profiling; multiple dysregulated miRNAs identified	No tissue-level miRNA studies available
Subchondral bone	EV-mediated miRNA transfer demonstrated	No studies available
Synovium	Several miRNAs linked to inflammation and disease severity	No studies available
Synovial fluid	Numerous circulating miRNAs identified as candidate biomarkers	No studies available
Circulation (serum/plasma)	Multiple studies reporting diagnostic and prognostic signatures	One exploratory study [5]

## Data Availability

Data sharing is not applicable. No new data were created or analyzed in this study.

## References

[1] Hunter D.J., Bierma-Zeinstra S. (2019). Osteoarthritis. Lancet.

[2] Tateuchi H., Koyama Y., Akiyama H., Goto K., So K., Kuroda Y., Ichihashi N. (2017). Daily Cumulative Hip Moment Is Associated with Radiographic Progression of Secondary Hip Osteoarthritis. Osteoarthr. Cartil..

[3] Loeser R.F., Collins J.A., Diekman B.O. (2016). Ageing and the Pathogenesis of Osteoarthritis. Nat. Rev. Rheumatol..

[4] Liu N., Ma Y., Gong W., Shao X., Shi T., Li L., Wu W., Chen X., Shi Y., Zhang P. (2025). Osteocyte-Derived Extracellular Vesicles Mediate the Bone-to-Cartilage Crosstalk and Promote Osteoarthritis Progression. Nat. Commun..

[5] Yamazaki A., Tomo Y., Eto H., Tanegashima K., Edamura K. (2022). A Pilot Study of microRNA Assessment as a Means to Identify Novel Biomarkers of Spontaneous Osteoarthritis in Dogs. Sci. Rep..

[6] Meeson R.L., Todhunter R.J., Blunn G., Nuki G., Pitsillides A.A. (2019). Spontaneous Dog Osteoarthritis—A One Medicine Vision. Nat. Rev. Rheumatol..

[7] Gregory M.H., Capito N., Kuroki K., Stoker A.M., Cook J.L., Sherman S.L. (2012). A Review of Translational Animal Models for Knee Osteoarthritis. Arthritis.

[8] Anderson K.L., O’Neill D.G., Brodbelt D.C., Church D.B., Meeson R.L., Sargan D., Summers J.F., Zulch H., Collins L.M. (2018). Prevalence, Duration and Risk Factors for Appendicular Osteoarthritis in a UK Dog Population under Primary Veterinary Care. Sci. Rep..

[9] Palazzo C., Nguyen C., Lefevre-Colau M.-M., Rannou F., Poiraudeau S. (2016). Risk Factors and Burden of Osteoarthritis. Ann. Phys. Rehabil. Med..

[10] Lauten S.D. (2006). Nutritional Risks to Large-Breed Dogs: From Weaning to the Geriatric Years. Vet. Clin. N. Am. Small Anim. Pract..

[11] Pearson-Ceol J. (2007). Literature Review on the Effects of Obesity on Knee Osteoarthritis. Orthop. Nurs..

[12] Loeser R.F., Goldring S.R., Scanzello C.R., Goldring M.B. (2012). Osteoarthritis: A Disease of the Joint as an Organ. Arthritis Rheum..

[13] Man G.S., Mologhianu G. (2014). Osteoarthritis Pathogenesis—A Complex Process That Involves the Entire Joint. J. Med. Life.

[14] Stannus O., Jones G., Cicuttini F., Parameswaran V., Quinn S., Burgess J., Ding C. (2010). Circulating Levels of IL-6 and TNF-α Are Associated with Knee Radiographic Osteoarthritis and Knee Cartilage Loss in Older Adults. Osteoarthr. Cartil..

[15] Shen J., Abu-Amer Y., O’Keefe R.J., McAlinden A. (2017). Inflammation and Epigenetic Regulation in Osteoarthritis. Connect. Tissue Res..

[16] Lepetsos P., Papavassiliou A.G. (2016). ROS/Oxidative Stress Signaling in Osteoarthritis. Biochim. Biophys. Acta (BBA)—Mol. Basis Dis..

[17] Ashraf S., Walsh D.A. (2008). Angiogenesis in Osteoarthritis. Curr. Opin. Rheumatol..

[18] Sellam J., Berenbaum F. (2010). The Role of Synovitis in Pathophysiology and Clinical Symptoms of Osteoarthritis. Nat. Rev. Rheumatol..

[19] Xu M., Ji Y. (2023). Immunoregulation of Synovial Macrophages for the Treatment of Osteoarthritis. Open Life Sci..

[20] Wood M.J., Miller R.E., Malfait A.-M. (2022). The Genesis of Pain in Osteoarthritis: Inflammation as a Mediator of Osteoarthritis Pain. Clin. Geriatr. Med..

[21] Shang X., Wang Z., Tao H. (2017). Mechanism and Therapeutic Effectiveness of Nerve Growth Factor in Osteoarthritis Pain. TCRM.

[22] Bartel D.P. (2004). MicroRNAs: Genomics, Biogenesis, Mechanism, and Function. Cell.

[23] Ha M., Kim V.N. (2014). Regulation of MicroRNA Biogenesis. Nat. Rev. Mol. Cell Biol..

[24] Schoenberg D.R., Maquat L.E. (2012). Regulation of Cytoplasmic mRNA Decay. Nat. Rev. Genet..

[25] Cheng P., Chen C., He H.B., Hu R., Zhou H.D., Xie H., Zhu W., Dai R.C., Wu X.P., Liao E.Y. (2013). miR-148a regulates osteoclastogenesis by targeting V-maf musculoaponeurotic fibrosarcoma oncogene homolog B. J. Bone Min. Res..

[26] Li Z., Hassan M.Q., Jafferji M., Aqeilan R.I., Garzon R., Croce C.M., van Wijnen A.J., Stein J.L., Stein G.S., Lian J.B. (2009). Biological Functions of miR-29b Contribute to Positive Regulation of Osteoblast Differentiation. J. Biol. Chem..

[27] Akhtar N., Rasheed Z., Ramamurthy S., Anbazhagan A.N., Voss F.R., Haqqi T.M. (2010). MicroRNA-27b Regulates the Expression of Matrix Metalloproteinase 13 in Human Osteoarthritis Chondrocytes. Arthritis Rheum..

[28] Mendell J.T., Olson E.N. (2012). MicroRNAs in Stress Signaling and Human Disease. Cell.

[29] Nims R., Palmer D.R., Kassab J., Zhang B., Guilak F. (2024). The Chondrocyte “Mechanome”: Activation of the Mechanosensitive Ion Channels TRPV4 and PIEZO1 Drives Unique Transcriptional Signatures. FASEB J..

[30] Lee W., Leddy H.A., Chen Y., Lee S.H., Zelenski N.A., McNulty A.L., Wu J., Beicker K.N., Coles J., Zauscher S. (2014). Synergy between Piezo1 and Piezo2 Channels Confers High-Strain Mechanosensitivity to Articular Cartilage. Proc. Natl. Acad. Sci. USA.

[31] Liu J., Wu X., Lu J., Huang G., Dang L., Zhang H., Zhong C., Zhang Z., Li D., Li F. (2021). Exosomal Transfer of Osteoclast-Derived miRNAs to Chondrocytes Contributes to Osteoarthritis Progression. Nat. Aging.

[32] Sun W., Zhao C., Li Y., Wang L., Nie G., Peng J., Wang A., Zhang L. (2016). Osteoclast-Derived microRNA-214-3p in Exosomes Inhibits Osteoblastic Bone Formation. Nat. Commun..

[33] Dai J., Dong R., Han X., Li J., Gong X., Bai Y., Kang F., Liang M., Zeng F., Hou Z. (2020). Osteoclast-Derived Exosomal Let-7a-5p Targets Smad2 to Promote the Hypertrophic Differentiation of Chondrocytes. Am. J. Physiol. Cell Physiol..

[34] Huang J., Li Y., Zhu S., Wang L., Yang L., He C. (2023). MiR-30 Family: A Novel Avenue for Treating Bone and Joint Diseases?. Int. J. Med. Sci..

[35] Ji Q., Xu X., Zhang Q., Kang L., Xu Y., Zhang K., Li L., Liang Y., Hong T., Ye Q. (2016). The IL-1β/AP-1/miR-30a/ADAMTS-5 Axis Regulates Cartilage Matrix Degradation in Human Osteoarthritis. J. Mol. Med..

[36] Li M., Gai F., Chen H. (2021). MiR-30b-5p Influences Chronic Exercise Arthritic Injury by Targeting Hoxa1. Int. J. Sports Med..

[37] Zhang X., Fang Z., Heng B.C., Hu X., Ge Z. (2025). Mitigating Oxidative Stress Enhances Cartilage Regeneration by Ameliorating Apoptosis of Cartilage Progenitor Cells in Adult Mice. Adv. Biol..

[38] Zhang L., Xu L., Wang Y., Zhang X., Xue T., Sun Q., Tang H., Li M., Cao X., Shi F. (2023). Histone Methyltransferase Setdb1 Mediates Osteogenic Differentiation by Suppressing the Expression of miR-212-3p under Mechanical Unloading. Cell. Signal..

[39] Tavallaee G., Rockel J.S., Lively S., Kapoor M. (2020). MicroRNAs in Synovial Pathology Associated with Osteoarthritis. Front. Med..

[40] Prasadam I., Batra J., Perry S., Gu W., Crawford R., Xiao Y. (2016). Systematic Identification, Characterization and Target Gene Analysis of microRNAs Involved in Osteoarthritis Subchondral Bone Pathogenesis. Calcif. Tissue Int..

[41] Iliopoulos D., Malizos K.N., Oikonomou P., Tsezou A. (2008). Integrative microRNA and proteomic approaches identify novel osteoarthritis genes and their collaborative metabolic and inflammatory networks. PLoS ONE.

[42] Shang X. (2025). Mechanosensitive miRNAs in Cartilage and Subchondral Bone Remodeling: Emerging Targets for Osteoarthritis Therapy. J. Inflamm. Res..

[43] Yu X.-M., Meng H.-Y., Yuan X.-L., Wang Y., Guo Q.-Y., Peng J., Wang A.-Y., Lu S.-B. (2015). MicroRNAs’ Involvement in Osteoarthritis and the Prospects for Treatments. Evid.-Based Complement. Altern. Med..

[44] Murata K., Yoshitomi H., Tanida S., Ishikawa M., Nishitani K., Ito H., Nakamura T. (2010). Plasma and Synovial Fluid microRNAs as Potential Biomarkers of Rheumatoid Arthritis and Osteoarthritis. Arthritis Res. Ther..

[45] Xie Y., Chen Y., Zhang L., Ge W., Tang P. (2017). The Roles of Bone-Derived Exosomes and Exosomal microRNAs in Regulating Bone Remodelling. J. Cell. Mol. Med..

[46] Swingler T.E., Wheeler G., Carmont V., Elliott H.R., Barter M.J., Abu-Elmagd M., Donell S.T., Boot-Handford R.P., Hajihosseini M.K., Munsterberg A. (2012). The Expression and Function of microRNAs in Chondrogenesis and Osteoarthritis. Arthritis Rheum..

[47] Withrow J., Murphy C., Liu Y., Hunter M., Fulzele S., Hamrick M.W. (2016). Extracellular Vesicles in the Pathogenesis of Rheumatoid Arthritis and Osteoarthritis. Arthritis Res. Ther..

[48] McAlinden A., Im G. (2018). MicroRNAs in Orthopaedic Research: Disease Associations, Potential Therapeutic Applications, and Perspectives. J. Orthop. Res..

[49] Beyer C., Zampetaki A., Lin N.-Y., Kleyer A., Perricone C., Iagnocco A., Distler A., Langley S.R., Gelse K., Sesselmann S. (2015). Signature of Circulating microRNAs in Osteoarthritis. Ann. Rheum. Dis..

[50] Ni Z., Zhou S., Li S., Kuang L., Chen H., Luo X., Ouyang J., He M., Du X., Chen L. (2020). Exosomes: Roles and Therapeutic Potential in Osteoarthritis. Bone Res..

[51] Ghorbaninejad M., Kamrani S., Ghorbaninejad Z., Hosseini S. (2025). Epigenetic Crosstalk in Bone and Cartilage Disorders: Emerging Role of Extracellular Vesicles. Biomed. Pharmacother..

[52] Valadi H., Ekström K., Bossios A., Sjöstrand M., Lee J.J., Lötvall J.O. (2007). Exosome-Mediated Transfer of mRNAs and microRNAs Is a Novel Mechanism of Genetic Exchange between Cells. Nat. Cell Biol..

[53] Arroyo J.D., Chevillet J.R., Kroh E.M., Ruf I.K., Pritchard C.C., Gibson D.F., Mitchell P.S., Bennett C.F., Pogosova-Agadjanyan E.L., Stirewalt D.L. (2011). Argonaute2 Complexes Carry a Population of Circulating microRNAs Independent of Vesicles in Human Plasma. Proc. Natl. Acad. Sci. USA.

[54] Tabet F., Vickers K.C., Cuesta Torres L.F., Wiese C.B., Shoucri B.M., Lambert G., Catherinet C., Prado-Lourenco L., Levin M.G., Thacker S. (2014). HDL-Transferred microRNA-223 Regulates ICAM-1 Expression in Endothelial Cells. Nat. Commun..

[55] Vickers K.C., Palmisano B.T., Shoucri B.M., Shamburek R.D., Remaley A.T. (2011). MicroRNAs Are Transported in Plasma and Delivered to Recipient Cells by High-Density Lipoproteins. Nat. Cell Biol..

[56] Mitchell P.S., Parkin R.K., Kroh E.M., Fritz B.R., Wyman S.K., Pogosova-Agadjanyan E.L., Peterson A., Noteboom J., O’Briant K.C., Allen A. (2008). Circulating microRNAs as Stable Blood-Based Markers for Cancer Detection. Proc. Natl. Acad. Sci. USA.

[57] Gallo A., Tandon M., Alevizos I., Illei G.G. (2012). The Majority of MicroRNAs Detectable in Serum and Saliva Is Concentrated in Exosomes. PLoS ONE.

[58] Lima J.F., Cerqueira L., Figueiredo C., Oliveira C., Azevedo N.F. (2018). Anti-miRNA Oligonucleotides: A Comprehensive Guide for Design. RNA Biol..

[59] Rupaimoole R., Slack F.J. (2017). MicroRNA Therapeutics: Towards a New Era for the Management of Cancer and Other Diseases. Nat. Rev. Drug Discov..

[60] van Rooij E., Kauppinen S. (2014). Development of microRNA Therapeutics Is Coming of Age. EMBO Mol. Med..

[61] Miyaki S., Lotz M.K. (2018). Extracellular Vesicles in Cartilage Homeostasis and Osteoarthritis. Curr. Opin. Rheumatol..

[62] Chorley B.N., Atabakhsh E., Doran G., Gautier J.-C., Ellinger-Ziegelbauer H., Jackson D., Sharapova T., Yuen P.S.T., Church R.J., Couttet P. (2021). Methodological Considerations for Measuring Biofluid-Based microRNA Biomarkers. Crit. Rev. Toxicol..

[63] Varvil M.S., Dos Santos A.P. (2023). A Review on microRNA Detection and Expression Studies in Dogs. Front. Vet. Sci..

[64] Witwer K.W. (2015). Circulating microRNA Biomarker Studies: Pitfalls and Potential Solutions. Clin. Chem..

[65] McDonald J.S., Milosevic D., Reddi H.V., Grebe S.K., Algeciras-Schimnich A. (2011). Analysis of Circulating microRNA: Preanalytical and Analytical Challenges. Clin. Chem..

[66] Zhou D., Li S., Wen J., Gong X., Xu L., Luo Y. (2008). Genome-Wide Computational Analyses of microRNAs and Their Targets from Canis Familiaris. Comput. Biol. Chem..

